# Are patients with inflammatory eye disease treated with systemic immunosuppressive therapy at increased risk of malignancy?

**DOI:** 10.1186/1869-5760-3-48

**Published:** 2013-05-31

**Authors:** William B Yates, Peter J McCluskey, Denis Wakefield

**Affiliations:** 1 Inflammation Research Unit, School of Medical Sciences, University of New South Wales, Sydney, NSW 2052, Australia; 2 Department of Ophthalmology, St Vincent's Hospital, Sydney, NSW 2052, Australia; 3 Save Sight Institute, Sydney University, Sydney, NSW 2001, Australia; 4 Ocular Inflammation Research Group, School of Medical Sciences, University of New South Wales, Sydney, NSW 2052; 5 Lowy Cancer Research Centre, Faculty of Medicine, University of New South Wales, Room 516, Level 5, Sydney, NSW 2052, Australia

**Keywords:** Immunosuppressive therapy, Incidence, Inflammatory eye disease, Malignancy, Neoplasia

## Abstract

The purpose of this study is to review the literature on the risk of malignancy in patients with inflammatory eye disease (IED) treated with systemic immunosuppressive (IS) therapy. Relevant databases in transplant medicine, autoimmune diseases and literature regarding uveitis and scleritis were reviewed. Literature with regards systemic IS therapy in transplant recipients and patients with autoimmune diseases revealed a significant increase in malignancies, especially non-melanocytic skin cancers and lymphomas. Studies of patients with IED were limited in number and scope, with no studies adequately evaluating the incidence of malignancy in these patients. Difficulties associated with the evaluation of the risk of malignancy associated with IS therapy in patients with IED include the heterogeneity of the disease and treatment regimens as well as the low frequency of IED, its variable severity and the lack of adequate long-term follow-up studies. Systemic IS therapy is an important therapeutic option in the treatment of patients with severe IED. A well-designed, comprehensive, multi-centre long-term follow-up study is required to evaluate the risk of malignancy in patients with specific IED diseases treated with defined systemic IS therapy. Until such evidence is available, we recommend the adoption of preventative strategies to help minimise the risk of malignancy in such patients.

## Review

### Introduction

The aims of therapy in patients with inflammatory eye disease (IED) are to control ocular inflammation, limit the progression of disease, preserve vision, maintain the quality of life and prevent local and systemic side effects. The treatment of severe IED with immunosuppressive (IS) therapy is a dilemma for the physician in determining whether the benefits of such therapy outweigh the risk of inducing other diseases, such as infection and malignancy. Literature regarding the use of IS therapy in the treatment of patients with autoimmune diseases indicates that these drugs induce secondary or *de novo* malignancies [1-3]. A crucial clinical question is if the manipulation of the immune system in patients with autoimmune diseases and transplant patients results in an increase in skin and other malignancies, does this mean that patients with IED treated with similar long-term IS therapy are also at such risk? Studies examining the risk of malignancies in transplant recipients, patients with human immunodeficiency virus (HIV) and autoimmune diseases show that suppressing the immune system predisposes the patients to carcinogenesis [4,5].

The importance of tumour immunosurveillance in humans has been inferred from clinical observations. The majority of evidence concerning the effects of IS therapy on the host immune response and tumour development is derived from studies of transplant recipients. The effectiveness of IS therapy in improving the outcome of organ transplantation has resulted in the development of malignancies and cardiovascular disease emerging as the major causes of patient mortality, rather than graft rejection as previously observed [1,3,6]. Solid organ transplant recipients given systemic immunosuppressive therapy require lifelong therapy. In contrast, patients with systemic inflammatory diseases are frequently treated with a number of immunosuppressive drugs, often concurrently and over a shorter time course, but nonetheless may still be predisposed to increased tumour risk [7].

Patients with severe IED are often treated with systemic immunosuppressive therapy. These patients either do not respond to topical or local therapies, have a type of IED not controlled by systemic corticosteroids or require aggressive therapy to prevent progressive vision loss [8]. The treatment of patients with chronic IED typically encompasses the use of corticosteroids with additional agents. This is primarily due to the significant side effects associated with long-term steroid therapy, and hence agents such as methotrexate (MTX), azathioprine (AZA), cyclosporine (CSA), mycophenolate mofetil (MMF) and, occasionally, alkylating agents (cyclophosphamide and chlorambucil) are given concurrently as steroid-sparing medications. Similarly, the efficacy of IS therapy in the treatment of a number of diseases such as ocular cicatricial pemphigoid [9], Behçet's disease [10] and Wegener granulomatosis [11] has been well documented.

Patients with idiopathic IED do not have an underlying systemic disease. Thus, these patients are a suitable group to examine the effect of immunosuppression and malignancy development in otherwise healthy individuals.

This review examines the evidence regarding the risk of malignancies in patients treated with systemic immunosuppressive therapy. The focus of the review is the evaluation of the evidence in the settings of transplant recipient, patients with autoimmune disease and IED. The aims are to evaluate the available literature and provide recommendations for further studies and prevention strategies to address this important clinical question.

### Materials and methods

A search of articles using the MEDLINE database and PubMed (National Library of Medicine) was performed to identify all relevant articles published in the area. Terms and phrases used for the search included immunosuppression, malignancy, transplantation, rheumatoid disease, autoimmune, uveitis, scleritis and ocular cicatricial pemphigoid. Articles were included if they were in English language and if full copies of the articles could be obtained. Articles were only included if they were from peer-reviewed journals. Articles were examined in reference to levels of evidence and recommendations set by the National Health and Medical Research Council. Articles were stratified on evidence strength dependent on study design, which included systematic reviews, randomised controlled trials (RCTs) and cohort study data. Review articles and editorials were also analysed and included if evidence was suitable for inclusion.

### Results

The incidence of malignancy in patients treated with IS therapy differed depending on the specific IS agents and the indication for therapy. Table 1 illustrates the incidence of malignancy in solid transplant recipients treated with a number of immunosuppressive agents. Table 2 highlights the literature relevant to systemic autoimmune diseases, and Table 3 summarises the literature regarding systemic immunosuppressive therapy in patients with IED.

**Table 1:** Studies which demonstrated an increased incidence of malignancy associated with systemic immunosuppressive therapy in transplant recipients

**Immunosuppressive therapy**	**Study**	**Risk of malignancy**	**References**
Azathioprine	1	162 patients with renal allografts treated with AZA (100 to 150 mg/day) found 22 NMSC developed at 3-year follow-up	[12]
	2	A comparison of AZA-based therapy (*n* = 3,139) and CSA-based therapy (*n* = 412) in transplant recipients found 1,255 skin malignancies developed in the AZA group (40%) compared to 90 in the CSA-based group (22%)	[13]
	3	12 malignancies, most commonly NMSC, developed in a group of transplant recipients of *n* = 287 during 12-month follow-up	[14]
	4	19 NMSC developed in 7 out of 51 kidney recipients (14%) treated with AZA + prednisone for between 4 and 45 months of treatment; dosing of AZA was kept at approximately 3 mg/kg/day for the duration after transplantation; 16 SCC, 1 BCC and 3 keratoacanthomas; conclusions suggested that tumours were increased in incidence and also prone to early reoccurrence	[15]
Mycophenolate mofetil	1	503 patients with renal allografts were randomised into 3 groups, 2 with varied MMF dosages (2 g, *n* = 173; 3 g, *n* = 164) and a group with AZA therapy (*n* = 166); 12 malignancies (lymphoma/LPD and non-skin carcinoma) were found in the group treated with 3 g MMF compared to 8 treated with 2 g MMF at 3-year follow-up	[12]
Cyclosporine	1	142 malignancies developed in 141 organ transplant recipients treated with cyclosporine, 41% lymphomas compared to 12% observed in the AZA, prednisone or prednisolone group at 20-year follow-up	[16]
	2	84 malignancies observed in 1,113 patients treated with combination CSA therapy (group 1) found a 1.99% cumulative risk of cancer 2 years post-transplant versus only 0.31% in the control (patients without CSA exposure or group 2); 43% of tumours observed (*n* = 38) were NMSC in group 1 compared to 27% in group not exposed to CSA, despite shorter time of exposure	[17]
	3	295 renal allograft recipients treated with AZA + prednisone (115) or AZA + prednisone + CSA (180) found 51 patients (19%) had at least 1 NMSC; it was found that the incidence of NMSC in AZA + prednisone was 29 per 1,000 person-years compared to 48 per 1,000 years in the group with additional CSA therapy	[18-21]

Follow-up of 3,823 patients treated with AZA, CYP or chlorambucil for >3 months found a 60-fold increase in NHL and 25-fold increase in SCC during the 5-year follow-up compared to the general UK and Australia population. SCC and BCC incidence in solid transplant recipients given systemic immunosuppressive therapy was demonstrated to be a four- to sevenfold increase in low sunshine exposure areas and a 20-fold increase in areas of high sun exposure compared to the general population. NHL incidence in transplant patients treated with immunosuppressive drugs show a 28- to 49-fold increase compared to age-matched controls.

**Table 2:** Studies which demonstrated an increased incidence of malignancy associated with systemic immunosuppressive therapy in autoimmune diseases

**Immunosuppressive therapy**	**Study**	**Risk of malignancy**	**References**
Cyclophosphamide	1	461 patients with RA treated with CYP found 5 bladder cancers developed during 5-year follow-up compared to the expected incidence for the general UK population (0.38)	[22]
	2	15 malignancies developed in 81 RA patients treated with CYP with a fourfold increase in the expected risk of malignancy compared to matched control RA patients not on cytotoxic therapy	[23]
	3	119 patients with RA treated with CYP were compared to 119 matched controls that found 37 malignancies in 29 patients and 16 malignancies in 16 patients in the control (*p* < 0.05); the major differences were in the number of bladder and NMSC observed (6 and 8, respectively) in the CYP-treated group and none in the control	[24]
Azathioprine	1	Analysis of data from 643 patients with RA found a 13-fold increase in NHL (whether treated with AZA or CYP)	[25,26]
	2	202 patients with RA treated with AZA compared to 202 RA patients without found a tenfold increase in NHL in patients treated with AZA and a fivefold increase in RA patients without therapy compared to the general population approximately 12-year follow-up	
Methotrexate	1	458 RA patients treated with MTX found a fivefold increase in NHL and threefold increase in melanoma compared to the general population with age standardisation; however, risk was increased with prior CYP exposure prior to MTX (2.5-fold increase)	[27]
Cyclosporine	1	In 1,252 patients with psoriasis followed up for 5 years, it was found that 47 patients (3.8%) developed malignancies; the standardised incidence ratio was 2.1 as compared with the general population; the study found a sixfold higher incidence in skin malignancies	[28]
Biologics	1	A RCT of 619 patients with RA treated with adalimumab and with previous MTX exposure found 4 adalimumab-treated patients developed NMSC, 1 non-Hodgkin's lymphoma and 1 adenocarcinoma	[29]
	2	In a JIA cohort of 7,812 treated with TNF inhibitors, an increased risk of malignancy in JIA patients compared to children without JIA was found; however, any increased risk of malignancy in patients treated with TNF inhibitors was not found	[30]
An epidemiologic study of non-transplant patients treated with systemic immunosuppression treated for greater than 3 months with AZA, CYP or chlorambucil found an increase in NHL by 11-fold, SCC by fivefold and carcinoma of the bladder by fourfold compared to the general population			[25]

**Table 3:** Studies which demonstrated an increased incidence of malignancy with systemic immunosuppressive therapy in IED

**Risk of malignancy**	**References**
8 malignancies were observed in 69 patients with ocular pemphigoid treated with cyclophosphamide and prednisone therapy (2 BCC, 2 SCC, 2 leukemias and 1 breast carcinoma)	[31]
537 patients treated with systemic corticosteroids and/or immunosuppressive therapy found no significant difference between groups treated with immunosuppressive (*n* = 330) and the control (*n* = 207), *p* > 0.90	[32]
46 patients treated with systemic immunosuppressive agents for uveitis were examined for malignancy incidence during a 5-year follow-up and compared to patients who only received corticosteroid therapy; 8 malignancies occurred in the experimental group in comparison to 2 in the control (*p* < 0.05)	[33]

#### Transplant recipients

Evidence from observational studies of transplant recipients provides significant evidence for the increased risk of malignancy associated with IS therapy. The emergence of effective systemic IS therapy for allograft recipients has seen a reduction in immunologic and non-immunologic graft rejection. However, the major contributor to mortality following solid organ transplantation is not graft rejection but malignancies and cardiovascular disease associated with IS therapy [1,34].

The Cincinnati Transplant Tumour Registry provided a great deal of evidence with regards to the effects of systemic IS therapy during the era (1980s and 1990s) when AZA and CSP were the major drugs used in post-transplantation patients. During 1988, it reported on 3,351 *de novo* malignancies in 3,320 patients treated with systemic IS therapy. Of this group, 3,139 were treated with AZA, and the majority of malignancies observed were skin (*n* = 1,255) (40%). Results of patients treated with CSP highlighted that the greatest proportion of the 412 tumours were lymphomas (*n* = 119) and skin cancers (*n* = 90) (29% and 22%, respectively) [34,35].

Skin malignancies, especially non-melanocytic skin cancer (NMSC), are the most common post-transplant tumours. Heart and renal transplant recipients are at an increased risk of squamous cell carcinoma (SCC) and basal cell carcinoma (BCC) with some studies quantifying the risk as 250 and 65 times higher than the general population, respectively [2,36]. Two theories have been proposed to explain this association including (1) solar-induced mutations coupled with immunosuppression reduces tumour surveillance [37] and (2) metabolites from certain IS agents such as AZA appear phototoxic and thus increase the skin's sensitivity to ultraviolet light damage [38]. Previous studies indicate that although solar radiation may be a major contributor of skin carcinogenesis, one increasingly important risk factor is the host's immune status [6,39,40]. To further illustrate this observation, one particular study analysed the distribution of NMSC in sites not exposed to solar radiation and found that the risk of NSMC and lip malignancies was disproportionate between the transplant group on systemic IS and the general population, regardless of whether sun-exposed or not (100- and 50-fold increase, respectively) [41].

#### Autoimmune diseases

Studies examining IS therapy in patients with RA are more directly comparable to studies of patients with IED. Patients with rheumatic diseases are commonly treated with similar immune-modulating agents as are patients with IED, including MTX, MMF, CSP and AZA [7,28].

This literature search revealed that a trend away from the use of alkylating agents (cyclophosphamide and chlorambucil) to anti-metabolites and T-cell inhibitors was initiated by findings of the effectiveness of these therapies and concerns regarding the risk of carcinogenesis resulting from an alkylating agent in the treatment of autoimmune diseases [22,42]. Kinlen found that 643 patients with RA treated with AZA and cyclophosphamide had a 13-fold increase of non-Hodgkin lymphoma (NHL) compared to the general population [25].

A number of studies suggested that RA itself increases the risk of malignancy regardless of therapy [43,44]. A large study of 46,000 patients illustrated a 2.7-fold increased risk of malignancy in patients with RA without IS therapy [45]. This study found a twofold higher incidence of leukaemia, lymphoma, NHL and myeloma in patients with RA compared to the Finnish cancer registry. However, patients with RA on IS therapy demonstrated a significantly greater increase (often documented up to 10- to 15-fold increase) in malignancy than in the study by Isomaki and associates [45]. Baltus and associates, for example, found a fourfold increase in malignancies compared to age-matched controls with RA, not on alkylating agent therapy [22].

A cohort study examining the risk of malignancies in patients with RA treated with MTX indicate that these patients are more susceptible to NHL, melanoma and lung cancer. This long-term follow-up (mean duration 9.3 years) demonstrated a fivefold higher risk of NHL and a threefold increase in melanoma [27]. Similarly, case reports examining the effect of MTX on patients with RA have concluded with similar findings, with some tumours regressing when therapy was withdrawn [46]. Comparable findings were established by Paul and associates in patients with psoriasis treated with cyclosporine, in whom found was a sixfold higher incidence of skin malignancies, mainly SCC, as compared to the general population [28]. It highlighted that patients treated for greater than 2 years with agents such as MTX, retinoids or another immunosuppressive drug had significantly greater risk than those treated with CSP alone (RR = 1.8, 2.9, 2.0, respectively).

Since the introduction of biologics, such as TNF-alpha inhibitors, there has been considerable debate with regard the risk of malignancy associated with these systemic IS agents. The majority of reliable data is derived from studies of patients with RA participating in RCTs and meta-analyses of such studies [4,29,47,48]. A meta-analysis examining adverse effects from infliximab and adalimumab studies found that malignancies were significantly more common in patients when given higher doses of TNF-inhibitors compared to those given low doses [48]. A recent retrospective cohort study by Beukelman and associates investigated 7,812 children with juvenile inflammatory arthritis (JIA) and found that children with this disease had a higher incidence of malignancy, with a standardised incidence ratio (SIR) of 4.4. In contrast to other research, it found that children treated with TNF inhibitors were not at a significantly increased risk of malignancy (SIR = 0) [30].

#### Inflammatory eye disease

There is limited data regarding the long-term sequelae of systemic IS therapy in the setting of IED. Murray and associates demonstrated that there was an increased risk of malignancy in patients treated with systemic IS (*n* = 46) therapy in comparison to corticosteroid therapy alone (*n* = 31). In this study, patients treated with corticosteroids were used as a control group as there is no known increased risk of malignancy associated with this drug [33]. The results demonstrated that eight malignancies occurred in the IS group of patients in comparison to two in the corticosteroid treated group (*p* < 0.05). Of particular note, the study established that the type of malignancies observed, namely, NHL, skin and cervical cancers, were similar to neoplasms that developed in patients treated with systemic IS therapy for allografts recipients and other autoimmune diseases. Lane et al. in, a larger study (*n* = 537), concluded that there was not a significantly increased risk of malignancy in patients with IED treated with IS therapy compared to patients treated with corticosteroid alone (*p* > 0.90) [32].

A large retrospective cohort study which examined cancer mortality risk in patients with IED treated with IS therapy found no increased risk of malignancy. Kempen and associates examined the overall mortality of patients with IED treated with systemic IS therapy. This study examined patients treated at five tertiary ocular inflammatory centres between 1979 and 2005 (*n* = 7957) and evaluated the overall mortality by identifiers on US National Death Index [49]. The conclusion drawn from this study indicated that patients with IED who were treated with systemic IS were not at an increased risk of death from malignancy. However, the study did suggest that TNF inhibitors doubled a patient's risk of malignancy. Crucially, the study did not address the prevalence of non-fatal malignancies in this cohort.

### Discussion

There is irrefutable evidence derived from studies of transplant recipients and patients with autoimmune diseases that IS therapy is associated with increased malignancy risk. The limited evidence of malignancy associated with systemic immunosuppressive therapy in patients with IED highlights that this clinical issue has not yet been appropriately addressed.

Data derived from transplant registries have strongly established the potential risk of malignancy that results from prolonged IS therapy in order to preserve graft function [39,50,51]. The ability to ascertain whether certain IS drugs are associated with a higher malignancy potential is also influenced by previous patient exposure to other IS therapies. These drugs are typically used in combination or consecutively as the introduction of newer IS agents, such as calcineurin inhibitors and biologics, became more widely used [34]. Long-term follow-up studies of transplant recipients revealed that neoplasms appeared on average 5 years after transplantation and the commencement of IS therapy [1,41]. Immunosuppressive therapy for allograft recipients differs significantly in regards to dosing and cumulative exposure when compared to patients treated for systemic inflammatory diseases. Similarly, the systemic inflammatory state induced by the allograft may drive carcinogenesis, exacerbated by the use of IS therapy [3]. Therefore, it would be naive to affirm the same risk of developing neoplasia without considering that there may be marked differences in risk between different groups of patients.

The literature on the risk carcinogenesis in patients with RA and other inflammatory diseases treated with IS drugs is divided. Numerous studies indicate an increased risk of malignancy with IS therapy, whilst other studies suggest that the increased risk is due to the disease itself (e.g. rheumatoid arthritis [7], psoriasis [28] and Sjögren's disease [52]). Isomaki et al. suggested that the baseline risk of malignancy in patients with RA might be due to chronic activation of the immune system, leading to an increased incidence of lymphoproliferative disorders. The majority of studies indicate that patients with either rheumatoid arthritis or psoriasis are at an increased risk of neoplasia; however, patients treated with systemic IS therapy have a significantly greater incidence of tumours [53-55]. A number of authors have factored in the baseline risk of neoplasia in patients and compared patients on IS therapy for RA and patients not on such therapy [56]. Other studies did not account for this baseline risk of malignancy in RA, which may inflate their malignancy incidence findings [57].

A well-designed study which accurately addresses the concern that systemic IS therapy in IED may predispose to malignancy has not been conducted, and there is limited literature specific to the long-term use of IS therapy in patients with IED. Studies that have examined this important issue have a number of significant shortcomings. The study conducted by Murray et al. established that there was an increased risk of malignancy; however, the findings are affected by a number of important factors. These include a small sample size (*n* = 87) and failure to account for pre-exposure risk of malignancy and not addressing IED with systemic disease associations in the analysis of the results [33]. The limited patient sample size resulted in insufficient statistical power for valid conclusions to be drawn. Lane and associates did not find an increased risk of malignancy in patients treated with IS agents, but it is possible that these results were affected by the relatively short-term follow-up period (mean 1.34 years). It is evident that long-term follow up (often >5 years) is necessary in studies of patients exposed to IS therapy in order to provide a more accurate clinical picture [1,58]. Kempen et al. suggested that systemic IS therapy does not increase the risk of cancer mortality in patients with IED [49]. The study, however, did not address the incidence of non-fatal neoplasia in patients treated with systemic IS therapy for IED. The type of malignancy, grade or histopathology for tumours was not obtained because the primary outcome of this study was defined as the cause of death on certificates identified on the US Death Index. Studies suggest that patients who develop malignancies associated with systemic IS therapy have tumours such as NMSC or lymphomas, which are treatable and may not directly increase mortality; however, they may have a significant impact on morbidity and the patient's quality of life [7,48,54]. Another important issue for ophthalmologists is the need for a regular, focussed and complete physical examination to ensure that the sites of common immunosuppression-related malignancies, such as skin, lymph nodes and cervix, are examined. This by necessity requires a careful systemic review and the assistance of other physicians, which may not be readily available in busy eye clinics.

More recently developed therapeutic agents, which are increasingly utilised in the treatment of ocular inflammation, target the activity of inflammatory cells and cytokine signalling. Limited long-term data exists on whether the use of these agents alters the body's ability to detect defective or pre-cancerous cells [59]. Data with regards the safety of biologics (e.g. TNF-α inhibitors) in the treatment of patients with IED are limited due to the recent use of these agents in this setting and a lack of long-term follow-up studies. A number of studies have highlighted the possibility of increased risk of malignancy although none of these studies involved a large patient sample with an appropriate long-term follow-up [60,61]. Similarly, issues related to pre-exposure to MTX and other systemic IS therapy may act as confounders on data reliability.

#### Recommendations for future studies

In order to properly address the question of whether the risk of malignancy increases in patients with IED treated with systemic IS therapy, a large collaborative multi-centre prospective cohort study with a long-term follow-up and regular careful systemic evaluation is required. The study design needs to incorporate methodology that addresses possible synergistic factors in carcinogenesis. Examples of such factors include past history and family history of malignancy, smoking status and occupation. In particular reference to NMSC, data on lifetime sun exposure and complexion should be obtained as in the study of Glover et al. [62]. This would evaluate whether an increase in malignancy in IED patients on systemic IS therapy is due predominantly to the immunosuppression or requires the addition of other factors and thus an indirect causation. Furthermore, comparison of patients with similar IED diseases treated with corticosteroid monotherapy as controls, compared to those treated with systemic IS therapy, would address issues with regard to the baseline malignancy risk in patients with IED. It could be argued that patients who are treated with systemic IS therapy may have more severe disease and thus are at significantly greater risk of carcinogenesis. Whilst this is true in the setting of autoimmune disease and transplant recipients, this same conclusion has not been demonstrated in patients with idiopathic IED.

An investigation of the malignancy risk associated with specific IED entities would provide crucial information. A number of tertiary IED centres would be required to achieve this aim by examination of a sufficiently large number of patients. Another important aspect of establishing the carcinogenesis potential of systemic IS therapy in patients with IED is to evaluate any dose–response relationship, which has not been conducted in patients with IED. This will ascertain whether higher dose IS therapy correlates with an increased risk of malignancy in patients with IED.

The large variety of systemic diseases associated with IED (e.g. Behçet's disease, sarcoidosis, Vogt-Kayangi-Harada disease, etc.) poses a problem in an investigation into malignancy risk. Patients with an associated systemic disease may have a significantly increased risk of malignancy compared to those with IED without an underlying disease. A large study, examining the common diseases associated with uveitis (with sufficient statistical power), is necessary to address this issue. Previous studies have tried to identify whether Behçet's disease is associated with an increased risk of malignant disease, but the available literature, consisting mainly of case reports, suggests no significant association [63,64]. An investigation into the baseline risk of neoplasia in patients with sarcoidosis also does not support such a correlation [65]. The baseline risk of malignancy has not been established for patients with IED, with or without systemic manifestations.

#### Prevention

A crucial aspect of the issue of the relationship between systemic IS therapy and cancer risk is how this can be minimised or prevented. Until it is proven beyond reasonable doubt that patients with IED treated with IS therapy are not at an increased risk of malignancy, it would seem prudent to adopt strategies to minimise such risk. Our suggestions for such an approach are outlined in Table 4. These are based in part on recommendations from the Kidney Disease: Improving Global Health Outcomes group. In relation to skin and lip cancers, it is recommended that patients should be educated about the risk of skin cancer, especially in subjects with a history of high levels of sun exposure and who are fair-skinned and/or have a history of previous NMSC [37]. All patients should be encouraged to practice regular self-examination and have annual skin and lip examinations by health professionals. Recommendations for non-skin cancer-related malignancies are the same as for cancer screening of the general population, with emphasis on cervical, breast, colon, renal and haematological examinations [66]. Immunisation against the human papilloma virus may also help reduce the incidence of cervical cancer in this population. Epidemiological studies of renal transplant patients indicate that the duration and intensity of IS therapy influence the incidence of post-transplant malignancies. Thus, modulation of IS therapy and minimising exposure to drugs known to have a high association with malignancy, such as cyclophosphamide, should be minimised and consideration given to the use of less oncogenic therapy, such as methotrexate and mTOR inhibitors, such as sirolimus [67]. There is evidence that mTOR inhibitors have a beneficial effect in terms of cancer regression in patients with post-transplant malignancy, and this will need to be confirmed in long-term studies.

**Table 4:** Strategy to reduce risk of systemic immunosuppressive therapy related malignancies

	**Strategy**
Pre-treatment	Careful pre-treatment evaluation of patients for presence of immunodeficiency (e.g. HIV), past or family history of malignancy and pre-malignant conditions (e.g. Bowen's disease, CIN of cervix, GIT polyps, leukoplakia of lip)
Dosages and therapy	Minimise dose and duration of immunosuppressive therapy (e.g. use of alkylating agents, such as cyclophosphamide for <12 months) - consider drugs with less oncogenic potential (e.g. MTX, mTOR inhibitors (sirolimus))
Education	Patient education - stop smoking, avoid excessive exposure to UV radiation, immunisation against HPV, regular self-examination (e.g. skin, lips, breast)
Follow-up	Regular and annual review - screening for common immunosuppressive therapy associated malignancies (e.g. skin, cervix, bladder, lymph nodes)

## Conclusion

The benefits of treatment of IED with IS drugs must be weighed against possible complications associated with such therapy [41]. The vision-saving benefits of the use of antimetabolites, calcineurin inhibitors and biologicals are thought to outweigh the risks [27]. A well-designed, comprehensive, multi-centre long-term follow-up study is required to evaluate the risk of malignancy in patients with specific IED diseases treated with defined systemic IS therapy. Such a study could provide valuable information that would influence the evaluation, investigation, prevention strategies, routine follow-up and screening of individuals. For example, patients at increased risk of malignancy, especially skin cancers, should receive advice regarding UV exposure and have regular skin examination. Immunisation against human papilloma virus could reduce the prevalence of cervical cancer. Similarly, long-term surveillance of patients treated with systemic IS therapy for IED should include regular systemic examination looking specifically for evidence of malignancy (skin, cervix, lymph nodes and blood). Until it has been ascertained as to whether or not there is an increased risk of malignancy associated with systemic IS therapy in patients with severe IED, increased vigilance is required to ensure that patients are regularly reviewed, appropriately and systematically examined, investigated and have long-term follow-up to detect and treat malignancies as early as possible.

## Competing interests

The authors report no proprietary or commercial interest in any product mentioned or concept discussed in this article. The authors declare that they have no competing interests.

## Authors' contributions

WBY conducted the initial literature search, wrote the initial draft and contributed critical appraisal. DW conceived the original idea for the review, provided direction for the manuscript and original ideas regarding prevention strategies. PMC provided original contributions to the discussion as well as providing direction and ideas regarding the literature in the field of inflammatory eye disease. The manuscript was a collaborative effort with all authors involved in the drafting and editing process. All authors read and approved the final manuscript.
